# Supplementary material for: Phagocytosis by an HIV antibody is associated with reduced viremia irrespective of enhanced complement lysis
Source: Nat Commun. 2022 Feb 3;13:662. doi: 10.1038/s41467-022-28250-7 (PMC8814042; doi:10.1038/s41467-022-28250-7)
Supplement: Supplementary file 1 — Supplementary Information [file 41467_2022_28250_MOESM1_ESM.pdf]

Phagocytosis by an HIV antibody is associated with reduced viremia irrespective of enhanced complement lysis

SUPPLEMENTARY INFORMATION

Supplementary Figure 1: Plasma 10E8v4 concentrations and neutralization titers in pharmacokinetic cohorts.

Supplementary Figure 2: Plasma 10E8v4 half-life and concentrations in the rectal mucosa in challenge cohorts.

Supplementary Figure 3: Post-acute PVL from all groups.

Supplementary Figure 4: Gating strategies for effector cell subsets.

Supplementary Figure 5: Gating strategy for T cell intracellular cytokine staining.

Supplementary Figure 6: 10E8v4 EFTAE can increase cell associated virus in the absence of complement-mediated lysis.

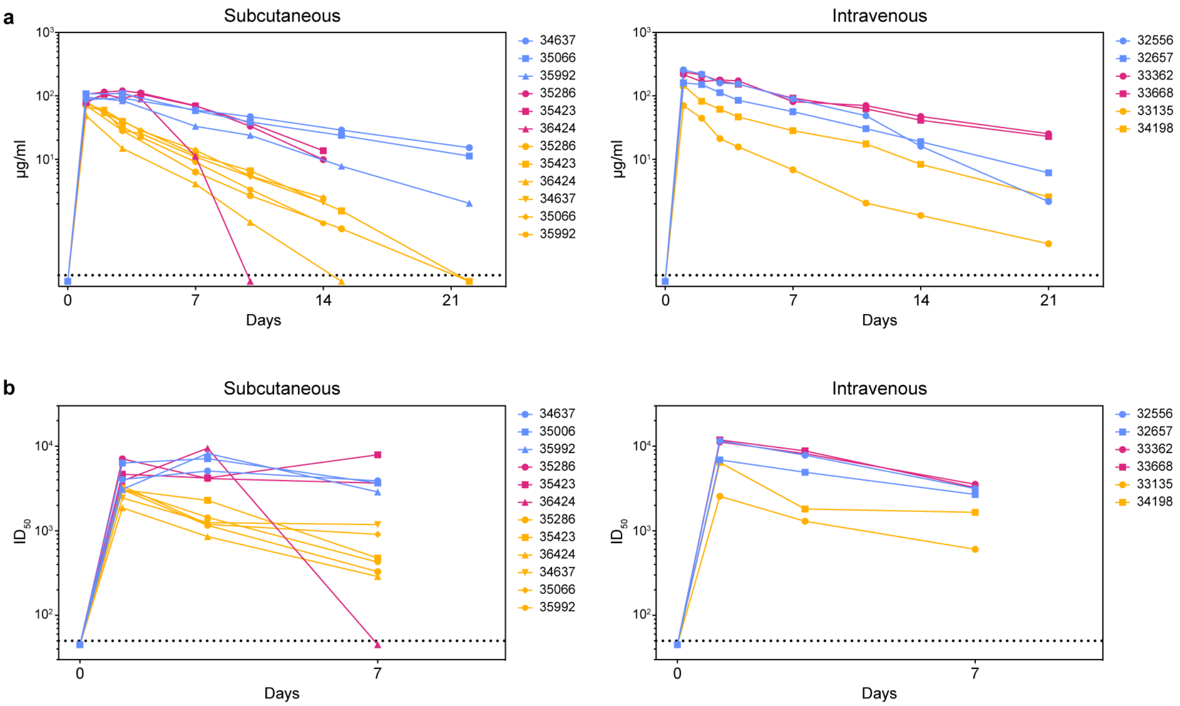

c

t1/2 pharmacokinetic cohorts				
	Variant	Animal	Plasma $t_{1/2}$	Average
Subcutaneous	Unmodified	34637	7.4	5.7
		35066	6.1	
		35992	3.6	
	LALA	35286	3.7	4.2
		35423	4.7	
		36424*	1.0	
	EFTAE	35286	2.3	2.3
		35423	2.3	
		36424*	1.7	
		34637	2.6	
		35066	2.6	
		35992	2.1	
Intravenous	Unmodified	32556	3.0	3.6
		32657	4.2	
	LALA	33362	6.4	6.1
		33668	5.8	
	EFTAE	33135	2.8	3.2
		34198	3.7	

*not included in average, has ADA

Supplementary Figure 1: Plasma 10E8v4 concentrations and neutralization titers in pharmacokinetic cohorts. Longitudinal plasma concentrations (a) or neutralization titer vs $HIV_{SF162P3}$ pseudovirus (b) of unmodified 10E8v4, 10E8v4 LALA, and 10E8v4 EFTAE in pharmacokinetic cohorts treated with 10 mg/kg and delivered either subcutaneously or intravenously as indicated. (c) Plasma antibody half-life for individual macaques. ADA: anti-drug antibodies.

a

	Variant	Animal	Plasma $t_{1/2}$	Average
Subcutaneous 5 mg/kg	Unmodified	29095	5.3	5.31
		32273	6.7	
		32311	6.1	
		33622	4.5	
		35151	5.1	
		35722	4.3	
	LALA	35781	4.5	5.27
		37171	4.7	
		26466	5.4	
		28264	8.7	
		37140	4.4	
		38067	3.9	
	EFTAE	35734	2.2	2.21
		35935	2.1	
		36330	2.1	
		36851	2.5	
		35472	1.6	
		36885	2.8	
Intravenous 10 or 20 mg/kg	Unmodified	30805	4.5	5.42
		31483	5.3	
		31486	5.9	
		33258	6.1	
	EFTAE	30192	1.9	2.16
		33817	3.4	
		34504	1.6	
		35400	1.7	

b

	NHP	Plasma (µg/ml)	Rectal Mucosa (µg/mg)	Plasma/Rectal Ratio	Rectal Mucosa Predicted
	30805	133.07	0.0126	10561	
	31483	104.06	0.0105	9910	
	31486	69.84	0.0098	7127	
	33258	77.89	0.0037	21051	
EFTAE 10+ mg/kg	30192	10.04	< 0.0010	nd	0.0008
	33817	8.95	< 0.0010	nd	0.0007
	34504	6.93	< 0.0010	nd	0.0006
	35400	6.58	< 0.0010	nd	0.0005
	29095	5.74	< 0.0010	nd	0.0005
	32273	5.45	< 0.0010	nd	0.0004
	32311	3.76	< 0.0010	nd	0.0003
	33622	3.91	< 0.0010	nd	0.0003
	35151	19.37	< 0.0010	nd	0.0016
	35722	13.41	< 0.0010	nd	0.0011
LALA 5 mg/kg	357181	4.77	< 0.0010	nd	0.0004
	37171	5.01	< 0.0010	nd	0.0004
	26466	3.54	< 0.0010	nd	0.0003
	28264	4.82	< 0.0010	nd	0.0004
	37140	33.37	< 0.0010	nd	0.0027
	38067	29.95	< 0.0010	nd	0.0025
EFTAE 5 mg/kg	35734	0.81	< 0.0010	nd	0.0001
	35935	0.96	< 0.0010	nd	0.0001
	36330	0.87	< 0.0010	nd	0.0001
	36851	1.02	< 0.0010	nd	0.0001
	35472	4.87	< 0.0010	nd	0.0004
	36885	4.02	< 0.0010	nd	0.0003

Supplementary Figure 2: Plasma 10E8v4 half-life and concentrations in the rectal mucosa in challenge cohorts. (a) Plasma antibody half-life for individual macaques in the challenge cohorts. (b) antibody concentrations in the plasma and rectal mucosa at 5 days post challenge (8 days post-delivery). In rectal mucosa, 10E8v4 was only detected in animals receiving 10+ mg/kg unmodified bNAb. An expected rectal concentration following the high dose unmodified average plasma/rectal ratio was calculated, and from it we infer that 10E8v4 EFTAE was not preferentially trafficking to the mucosa, because if so, it would have been above the limit of detection in the high dose EFTAE group.

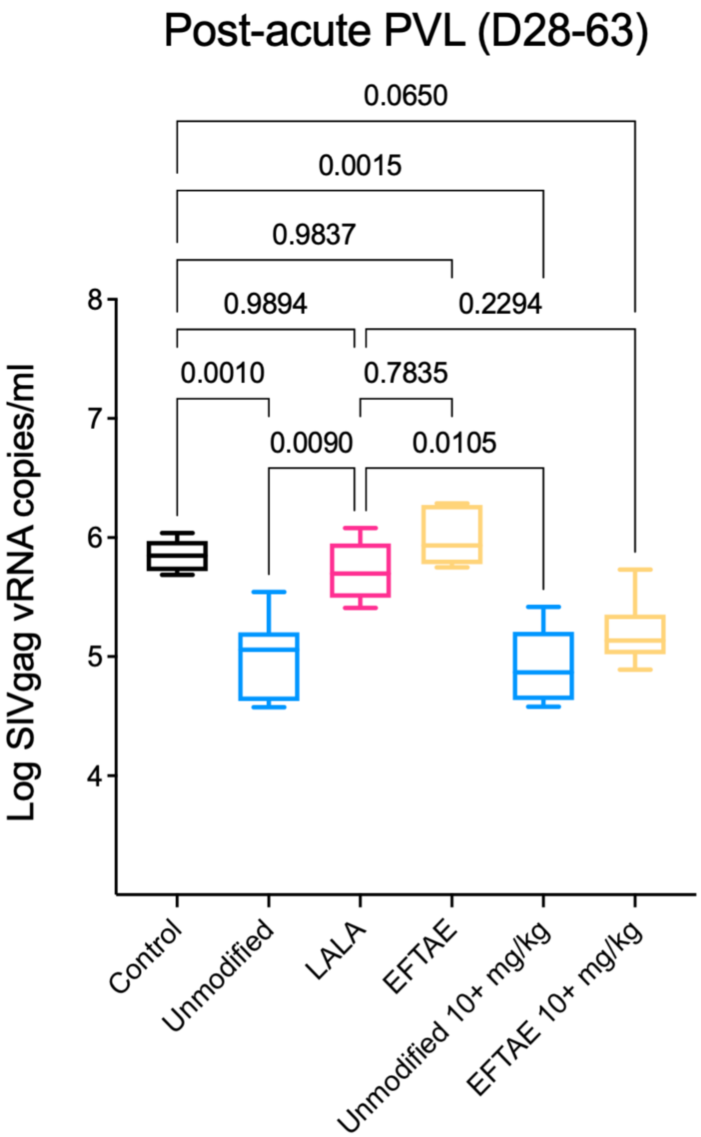

Supplementary Figure 3: Post-acute PVL from all animal cohorts were analyzed by one-way ANOVA followed by Tukey's post-hoc comparison between groups. Boxes in box and whisker plots extend from 25-75 percentiles with a line at median and whiskers extending to min-max values. P values for comparisons between groups without effector function present (LALA and Control) are provided. Statistical comparisons were performed using a two-way ANOVA followed by Tukey's post-hoc comparison between groups. Viral loads presented in main text Figures 4d and 5e were analyzed using $\log_{10}$ transformed data. All <0.05 adjusted p values are shown as well as non-significant p values between comparisons mentioned in the text. Data and analysis are derived from n=6 animals per group (5 mg/kg); n=4 animals per group (10+ mg/kg). Group colors are consistent throughout the manuscript and color key is shown in main text Figure 7. Statistical significance was determined at the significant alpha level of 0.05 and performed in GraphPad Prism 9. Data shown are representative of at least two independent experiments. Source data are provided in the Source Data file associated with this manuscript.

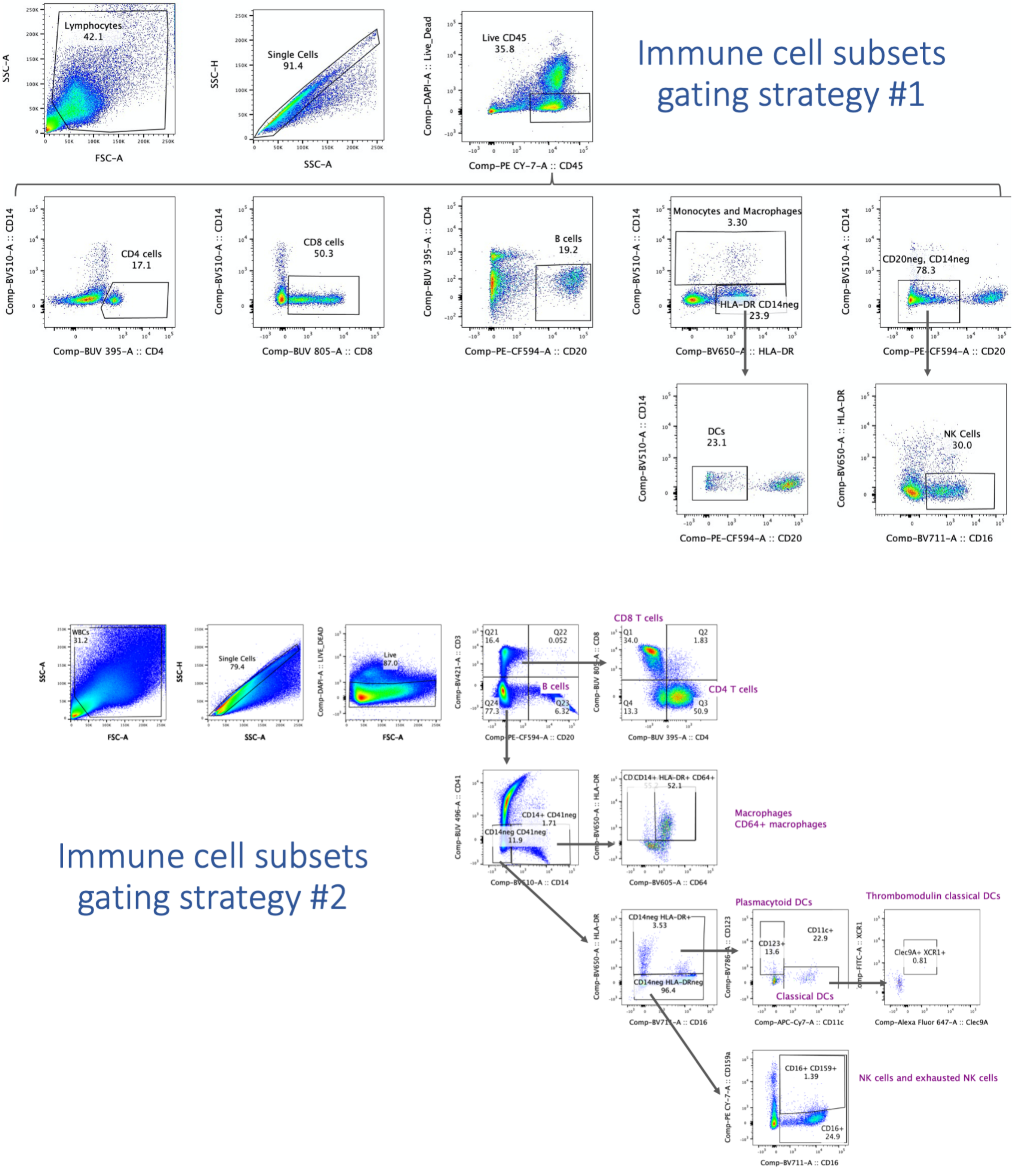

Supplementary Figure 4: Gating strategy for effector cell subsets. Two flow panels and gating strategies were employed to examine effector cell subsets. Strategy #1 (upper scheme) was used to report data in Figure 7a. No significant differences were observed in specific subsets interrogated in strategy #2 (lower scheme) between groups.

T cell ICS gating strategy

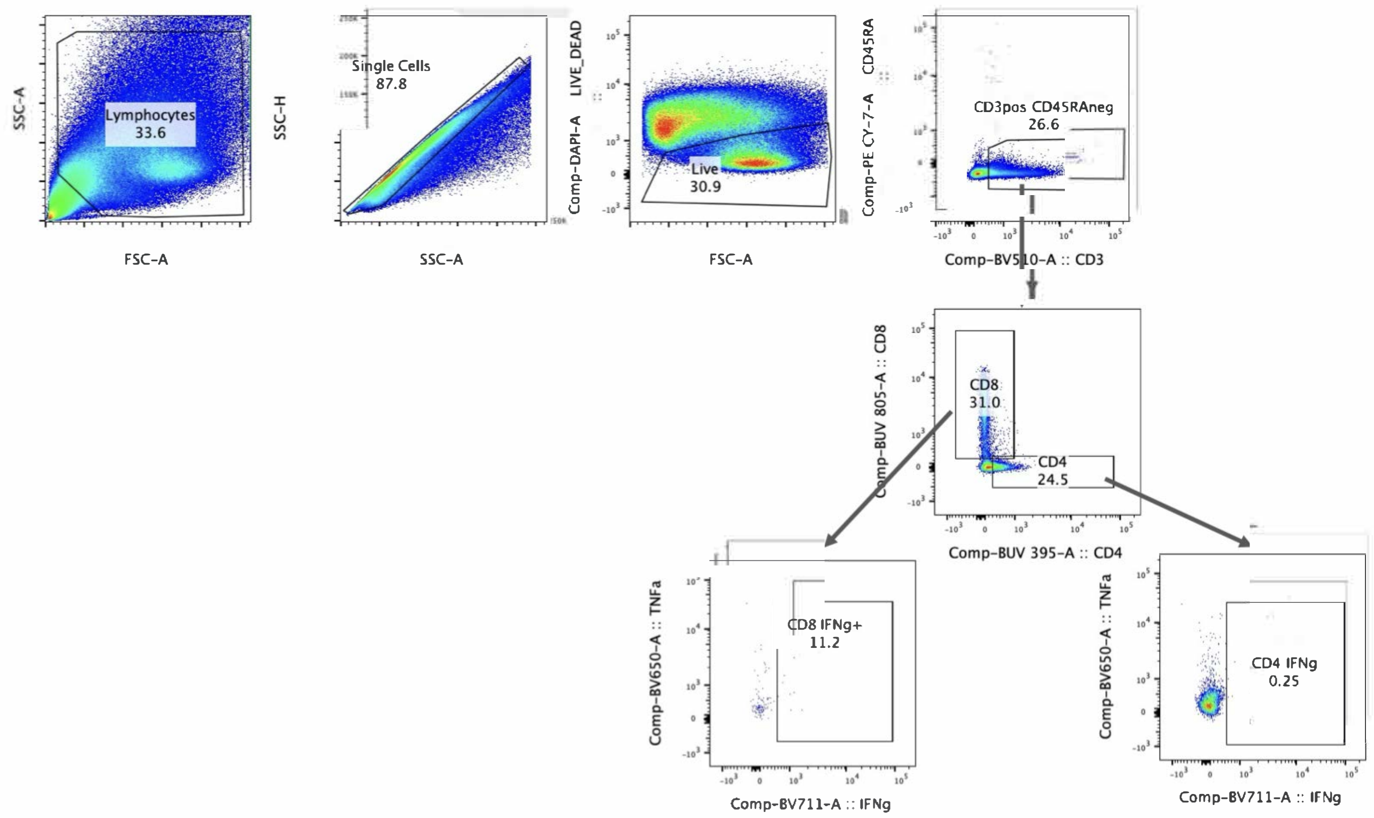

Supplementary Figure 5: Gating strategy for T cell intracellular cytokine staining. Specific T cells in Figure 7e and 7f were measured as the IFN- + subset following stimulation with GAG and Env peptide pools minus those following mock stimulation as determined by the gating strategy above. Data analysis is shown in Figure 7 of the main text.

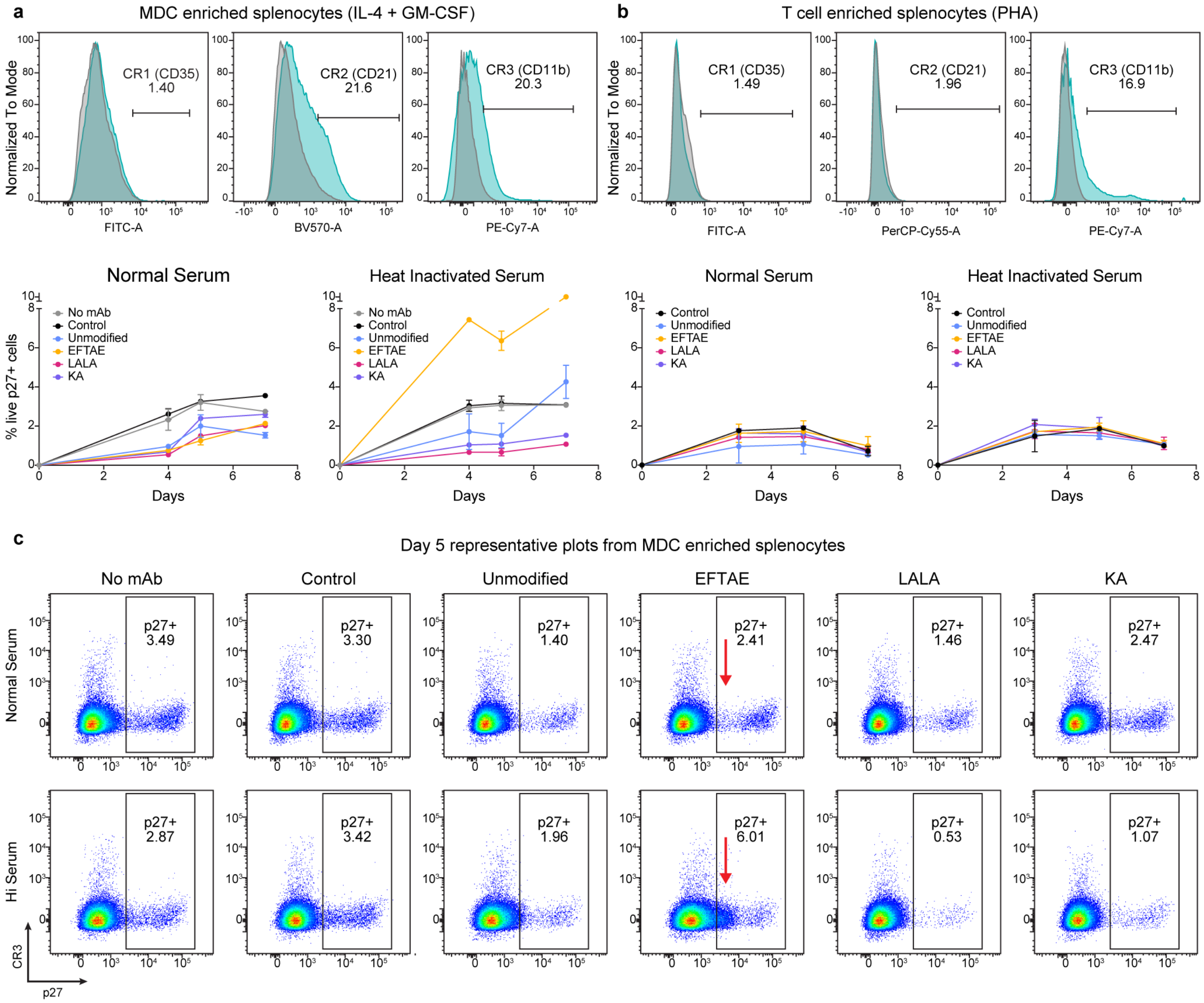

Supplementary Figure 6: 10E8v4 EFTAE can increase cell associated virus in the absence of complement-mediated lysis. Naïve rhesus splenocytes were harvested and stimulated with either (a) IL-4 and GM-CSF to enrich monocyte derived dendritic cells expressing complement receptors or (b) PHA to enrich T cells. Stimulated cells were cultured for 7 days, then incubated with 5 µg/ml of the indicated 10E8v4 variant. Separately, 700 ng/ml $SHIV_{SF162P3}$ was incubated in 20% normal or heat inactivated rhesus serum immediately prior to spinoculation of 10E8v4 treated cells. The resulting percentage of infected cells was monitored by flow cytometry for intracellular p27. (c) Representative scatter plots of flow staining five days following spinoculation. Each antibody treatment was performed in duplicate and results are representative of three independent experiments. Graphed data shown in a, b is mean +/- se.
