# Supplementary material for: Phagocytosis by an HIV antibody is associated with reduced viremia irrespective of enhanced complement lysis
Source: Nat Commun. 2022 Feb 3;13:662. doi: 10.1038/s41467-022-28250-7 (PMC8814042; doi:10.1038/s41467-022-28250-7)
Supplement: Supplementary file 3 — Reporting Summary [file 41467_2022_28250_MOESM3_ESM.pdf]

nature portfolio

DBPR
Corresponding author(s): NCOMMS-21-29317B
Last updated by author(s): Jan 10, 2022

Reporting Summary

Nature Portfolio wishes to improve the reproducibility of the work that we publish. This form provides structure for consistency and transparency in reporting. For further information on Nature Portfolio policies, see our Editorial Policies and the Editorial Policy Checklist.

Statistics

For all statistical analyses, confirm that the following items are present in the figure legend, table legend, main text, or Methods section.

n/a	Confirmed	
☐	☒	The exact sample size (*n*) for each experimental group/condition, given as a discrete number and unit of measurement
☐	☒	A statement on whether measurements were taken from distinct samples or whether the same sample was measured repeatedly
☐	☒	The statistical test(s) used AND whether they are one- or two-sided<br>*Only common tests should be described solely by name; describe more complex techniques in the Methods section.*
☒	☐	A description of all covariates tested
☐	☒	A description of any assumptions or corrections, such as tests of normality and adjustment for multiple comparisons
☐	☒	A full description of the statistical parameters including central tendency (e.g. means) or other basic estimates (e.g. regression coefficient) AND variation (e.g. standard deviation) or associated estimates of uncertainty (e.g. confidence intervals)
☐	☒	For null hypothesis testing, the test statistic (e.g. F, t, r) with confidence intervals, effect sizes, degrees of freedom and P value noted<br>*Give P values as exact values whenever suitable.*
☒	☐	For Bayesian analysis, information on the choice of priors and Markov chain Monte Carlo settings
☐	☒	For hierarchical and complex designs, identification of the appropriate level for tests and full reporting of outcomes
☐	☒	Estimates of effect sizes (e.g. Cohen's d, Pearson's r), indicating how they were calculated

Our web collection on statistics for biologists contains articles on many of the points above.

Software and code

Policy information about availability of computer code

Data collection	No software was used for data collection
Data analysis	Statistical tests were performed in GraphPad Prism version 9 and checked by OHSU biostatisticians.

For manuscripts utilizing custom algorithms or software that are central to the research but not yet described in published literature, software must be made available to editors and reviewers. We strongly encourage code deposition in a community repository (e.g. GitHub). See the Nature Portfolio guidelines for submitting code & software for further information.

Data

Policy information about availability of data

All manuscripts must include a data availability statement. This statement should provide the following information, where applicable:
- Accession codes, unique identifiers, or web links for publicly available datasets
- A description of any restrictions on data availability
- For clinical datasets or third party data, please ensure that the statement adheres to our policy

There is no restrictions on data availability. The complete (raw) data tables will be provided with the manuscript.

Field-specific reporting

Life sciences study design

All studies must disclose on these points even when the disclosure is negative.

Sample size	For the initial 24 macaques treated with 5 mg/kg dosing, macaque 4 treatment groups of N=6 were chosen to test Fc functionality within budget constraints. A follow-up study with doses of 10+ mg/kg was then performed on 2 of the treatment groups with N = 4 per group and N=8 total to provide further clarity on Fc contributions.
Data exclusions	No data was excluded
Replication	Each macaque antibody treatment group was run in two or three different animal cohorts (sequentially, not in parallel). Key measures of viral load, antibody binding, neutralization, Fc effector functions, etc were assayed in duplicate or triplicate, often with serial dilutions. In many cases, experiments were repeated to confirm accuracy of results.
Randomization	Macaques were divided as evenly as possible into each treatment group based on age, weight, sex, and body composition scores (bsc).
Blinding	Investigators were immediately instrumental in designing experiments and managing the project, hence animal groups and experimental assay results was generally not blinded. However, plasma and tissue samples provided to the ONPRC Virology Core for quantifying virus loads were blinded.

Reporting for specific materials, systems and methods

We require information from authors about some types of materials, experimental systems and methods used in many studies. Here, indicate whether each material, system or method listed is relevant to your study. If you are not sure if a list item applies to your research, read the appropriate section before selecting a response.

Materials & experimental systems

n/a	Involved in the study	
☐	☒	Antibodies
☐	☒	Eukaryotic cell lines
☒	☐	Palaeontology and archaeology
☐	☒	Animals and other organisms
☒	☐	Human research participants
☒	☐	Clinical data
☒	☐	Dual use research of concern

Methods

n/a	Involved in the study	
☒	☐	ChIP-seq
☐	☒	Flow cytometry
☒	☐	MRI-based neuroimaging

Antibodies

Antibodies used	Expression plasmid for the parental antibody 10E8v4 was obtained via MTA from the NIH Vaccine Research Center. Production and purification of the mAbs used in NHP were provided by Scripps Antibody Production Core in a fee-for service agreement. Fc variants of 10E8v4 were generated by the authors
Validation	As this study was a investigation into antibody functionality, mAb 10E8v4 and variants were extensively characterized/validated in Figures 1 and 2 of the manuscript. QC of the produced mAbs for purity and lack of endotoxin were performed by Scripps Antibody Core.

Eukaryotic cell lines

Policy information about cell lines

Cell line source(s)	TZM-bl cells were obtained from the ATCC. For ADCC assays, CD4+CCR5+ NKR24 and KHYG-1 NK cell lines were provided by David Evans. TZM-bl cells with FcRs were obtained by the NIH Aids Reagents Program (ARP-11796, ARP-11797, ARP-11798)
Authentication	Cell lines were not directly authenticated, but positive control assay conditions were verified to match that of historical controls.
Mycoplasma contamination	The cell lines were not tested for mycoplasma contamination.
Commonly misidentified lines (See ICLAC register)	*Name any commonly misidentified cell lines used in the study and provide a rationale for their use.*

Animals and other organisms

Policy information about studies involving animals; ARRIVE guidelines recommended for reporting animal research

Laboratory animals	Adult male and female rhesus macaques of Indian origin double negative for MHC alleles Mamu-B*08 and Mamu-B*17 were used in

	this study
Wild animals	This study did not use wild animals.
Field-collected samples	Samples were collected by veterinary staff at the Oregon National Primate Research center under the oversight of the attending veterinarian.
Ethics oversight	All animal experiments were conducted under the oversight of the institutional animal care and use committee (IACUC) at Oregon Health and Science University.

Note that full information on the approval of the study protocol must also be provided in the manuscript.

Flow Cytometry

Plots

Confirm that:

- [x] The axis labels state the marker and fluorochrome used (e.g. CD4-FITC).
- [x] The axis scales are clearly visible. Include numbers along axes only for bottom left plot of group (a 'group' is an analysis of identical markers).
- [x] All plots are contour plots with outliers or pseudocolor plots.
- [x] A numerical value for number of cells or percentage (with statistics) is provided.

Methodology

Sample preparation	Detailed in the text for the specific assay performed and reported.
Instrument	FACSymphony A5 cell analyzer (BD Biosciences)
Software	FlowJo version 10.8
Cell population abundance	Sorting was not performed for this manuscript.
Gating strategy	Detailed in the text for the specific assay performed and reported.

- [x] Tick this box to confirm that a figure exemplifying the gating strategy is provided in the Supplementary Information.
